# Serum CXCL5 level is associated with tumor progression in penile cancer

**DOI:** 10.1042/BSR20202133

**Published:** 2021-01-27

**Authors:** Miao Mo, Yangle Li, Xiheng Hu

**Affiliations:** Department of Urology, Xiangya Hospital, Central South University, Changsha, Hunan 410008, P.R. China

**Keywords:** CXCL5, CXCR2, penile cancer, prognosis, tumor progression

## Abstract

Chemokine (C-X-C motif) ligand 5 is an important regulator of tumor progression in many cancers, and could serve as potential serum cancer biomarker. Our initial analysis identified CXCL5 as a cancer-related gene highly expressed in PC. Patients with PC exhibited markedly higher preoperative serum CXCL5 levels compared with that in healthy individuals (*P*<0.001). The area under the curve (AUC) was 0.880 with the sensitivity of 84.0%, and specificity of 80.4% to distinguish PC. Serum CXCL5 levels were also significantly decreased following tumor resection in patients with PC (*P*=0.001). Preoperative serum CXCL5 level was significantly associated with clinicopathological characteristics including T stage (*P*=0.001), nodal status (*P*<0.001), and pelvic lymph node metastasis (*P*=0.018). Cox regression analysis showed that serum CXCL5 level could serve as an independent prognostic factor for disease-free survival with a HR of 6.363 (95% CI: 2.185–18.531, *P*=0.001). CXCL5 and its receptor CXCR2 exhibited correlated expression pattern in PC tissues. Differential CXCL5 expression was observed in normal penile tissues, PC cell lines, and their culture supernatants. Furthermore, knockdown of CXCL5 or CXCR2 expression markedly suppressed malignant phenotypes (cell proliferation, clonogenesis, apoptosis escape, migration, and invasion), attenuated STAT3 and AKT signaling, and reduced MMP2/9 secretion in PC cell lines. In conclusion, our findings revealed that serum CXCL5 level might serve as a potential diagnostic and prognostic cancer biomarker for penile cancer. Autocrine CXCL5/CXCR2 signaling might activate multiple downstream oncogenic signaling pathways (STAT3, AKT, MMP2/9) to promote malignant progression of PC, which may warrant further investigation in the future.

## Introduction

Penile cancer (PC) is a rare cancer in developed countries; however, its incidence rate is much higher in some regions of South America, Asia, and Africa [1]. Despite recent progress in multimodal therapies, the clinical outcome of PC remains unsatisfactory, as the survival of patients with PC has not improved during the last two decades [2]. Serum cancer biomarkers, such as carcinoembryonic antigen (CEA), cancer antigen (CA)-125, and CA-15-3, have been beneficial in the diagnosis of cancer and disease monitoring. However, these serum cancer biomarkers are not beneficial in PC [3–5]. Squamous cell carcinoma antigen (SCC) levels have been associated with PC tumor burden; although it could not predict the clinical outcome [6]. On the other hand, overexpression of p53 or Ki-67 has been associated with tumor progression of PC, yet these biomarkers are not used in a clinical setting [7–9].

Chemokines may play an important role in the development of tumorigenesis in numerous types of cancer [10,11]. Aberrant expression of some chemokines, such as CCL5, CXCL1, CXCL8 and CXCL13, has been detected in several types of cancer [12–15]. C-X-C motif chemokine ligand 5 (CXCL5) is an important chemokine secreted by immune cells, such as monocytes and T lymphocytes [16]. Recent studies indicated that CXCL5 is aberrantly expressed in >14 different types of cancer, including hepatocellular carcinoma, prostate cancer, pancreatic cancer, and gastric cancer [17]. Moreover, CXCL5 expression has been found to be associated with the degree of malignancy, metastatic potential, and degree of inflammatory infiltration in numerous types of cancer. In gastric cancer, CXCL5 is associated with late stages of the disease [18]. The expression levels of CXCL5 in colorectal cancer tissues are also found to be associated with malignant phenotypes of prostate cancer [19]. In hepatocellular carcinoma, CXCL5 was found to promote neutrophil infiltration and indicates poor prognosis [20]. In pancreatic cancer, CXCL5 is overexpressed in cancer tissues and is significantly associated with poorer tumor differentiation, advanced clinical stage and shorter patient survival [21]. Thus, the aim of the present study was to examine the expression of CXCL5 in PC and to evaluate the usefulness of serum CXCL5 levels as a potential cancer biomarker for PC.

## Materials and methods

### Patient characteristics

A total of 81 patients were included in the present retrospective study, and underwent surgery and were diagnosed with PC between 2016 and 2018 at Xiangya Hospital, Central South University (Hunan, China). Patients who received chemotherapy or brachytherapy previously were excluded from the study. The serum samples of 46 healthy male control were obtained from the Health Examination Center (Xiangya Hospital, Central South University, Hunan, China) and provided informed consent. TNM staging was performed according to the American Joint Committee on Cancer guidelines, 8th edition [22]. The clinical parameters of the patients with PC included age, T stage, nodal status, histological subtype, pathological grade and body mass index (BMI), as well as phimosis.

### Reagents and cell lines

The primary antibodies against CXCL5, CXCR2, phosphorylated (p)-STAT3 (Tyr705), STAT3, p-ERK1/2 (Thr202/Tyr204), ERK1/2, p-AKT (Ser473), AKT, and β-actin were purchased from Abcam. The human Penl1, Penl2, 149RCa, and LM156 PC cell lines were kindly provided by Prof. Hui Han (Department of Urology, Cancer Hospital, Sun Yat-Sen University) [23]. The cell lines were cultured in Dulbecco’s modified Eagle’s medium supplemented with 10% fetal bovine serum as previously described [23]. Lentiviral short hairpin (sh)RNAs targeting shCXCL5 or shCXCR2 were purchased from GeneCopoeia Inc, and were used as previously described [24,25].

### ELISA assay

All the blood samples were collected 1 day prior to (preoperative) or on day 28 following surgery (postoperative). Serum samples were separated and stored at −80°C for further analysis. Serum CXCL5 levels were measured using CXCL5 ELISA Kit (RayBiotech, Inc.), according to the manufacturer’s protocol.

### Cell growth analysis

Cell growth was measured using the Cell Counting Kit-8 (CCK-8) assay as previously described [24]. The CCK-8 absorbance (optical density OD_450_) was measured using a MK3 microplate reader (Thermo Fisher Scientific, Inc.).

### Clonogenic assay

The clonogenic assay was conducted to measure the clonogenic potential of PC cells as previously described [25]. Briefly, PC cells were seeded in 6-cm culture dishes, and cultured for 12 days. The number of colonies (contains >50 cells) was counted.

### Wound healing assay

Cell migration ability was measured using a wound healing assay as previously described [26]. Briefly, PC cells were cultured to confluency, and subsequently a uniform wound was created for each experiment group. The distance between the wound sides was measured immediately following the creation of the wound and after 24 h.

### Transwell invasion assay

The cell invasion ability was measured using the transwell chamber as previously described [26]. The invaded cells, on the bottom surface of the 8-µm pore membrane were stained using 0.2% Crystal Violet, eluted by acetic acid and measured with a MK3 microplate reader (Thermo Fisher Scientific, Inc.) at 570 nm.

### Western blot analysis

The cell lysates were prepared using RIPA lysis buffer and the remaining Western blot analysis used performed as previously described [26]. The protein blots were visualized using an ECL kit (Abcam).

### Bromodeoxyuridine (BrdU) incorporation assay

The bromodeoxyuridine (BrdU) incorporation assay was used to assess the proliferative potential of PC cells as previously described [27]. Briefly, PC cells were plated (4 × 10^3^ cells/well) into 96-well plate and incubated for 72 h. Following incubation, PC cells were labeled with 10 µM BrdU for 2 h and the incorporated BrdU was detected using a BrdU Assay Kit (Abcam).

### Caspase-3 activity assay

Cellular apoptosis was detected using Caspase-3 Colorimetric Assay Kit (Abcam) as previously described [27]. Briefly, cells (5 × 10^5^) were lysed on ice for 10 min and centrifuged at 10,000 × ***g*** for 1 min. Enzyme reactions were performed on the resulting supernatants in 96-well flat-bottom microplates, using 50 µl cell lysate (100 µg of total protein) for each reaction mixture. The OD was measured at 405 nm using a Multiskan MK3 microplate reader (Thermo Fisher Scientific, Inc.).

### Immunohistochemistry (IHC)

Archived paraffin-embedded normal penile tissues (*n*=30) and PC tissues (*n*=40) were collected for IHC staining. These patients had undergone surgery and were diagnosed with PC between 2017 and 2018 at Xiangya Hospital, Central South University (Hunan, China). IHC was performed as previously described [28]. Antigen-antibody reactions (dilution for CXCL5, 1:50; dilution for CXCR2, 1:100) were visualized by exposure to 3,3-diaminobenzidine and hydrogen peroxide chromogen substrate (DAKO; Agilent Technologies, Inc.). Positive CXCL5 staining (≥30%) was regarded as high expression [24].

### GEO dataset

GEO dataset GSE57955 could be downloaded from NCBI GEO website (https://www.ncbi.nlm.nih.gov/geo/query/acc.cgi?acc=GSE57955). Gene expression data were analyzed as described previously [24]. Genes with a mean log_2_ signal ratio (penile cancer/normal tissue pool) of ≥ 1.0 and ≤ −1.0 within a 99% confidence interval were considered differentially expressed.

### Statistical analysis

Statistical analyses were performed using SPSS v16.0 software. The serum CXCL5 levels between two groups were compared using Mann–Whitney tests. The pre- and post-operative serum CXCL5 levels were compared using a Wilcoxon rank sum test. The optimal cut-off value of preoperative serum CXCL5 was determined based on receiver-operating characteristic (ROC) analysis with reference to cancer recurrence. Kaplan–Meier curves of disease-free survival (DFS) were plotted, and survival in the groups was compared using a log-rank test. The prognostic factors that influence DFS were identified using univariable and multivariable Cox regression analysis. *P*<0.05 was considered to indicate a statistically significant difference.

## Results

### CXCL5 is highly expressed in PC tissues

**Figure 1:**
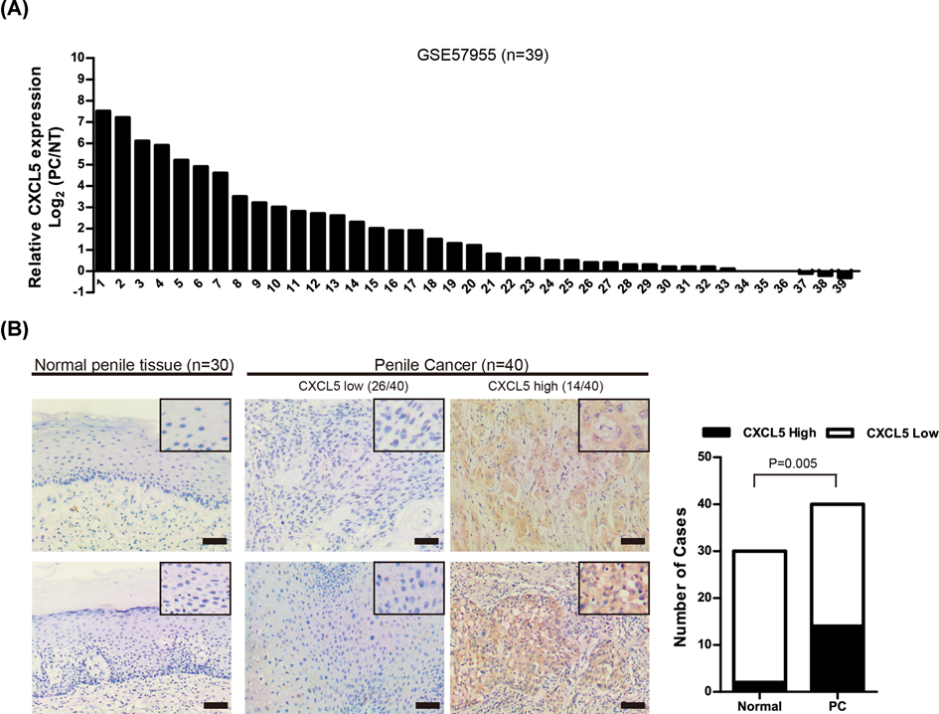
CXCL5 is highly expressed in PC tissues (**A**) Waterfall plot of CXCL5 expression in GSE57955 dataset (*n*=39). (**B**) Immunohistochemistry on CXCL5 expression in normal penile tissues (*n*=30) and PC (*n*=40) tissues. Representing micrographs showed high or low CXCL5 expression in two PC cases, respectively; bars: 100 µm.

The mRNA expression of CXCL5 in PC was analyzed in public GEO dataset GSE57955 (*n*=39). CXCL5 was highly expressed in PC with reference to normal tissue pool (NT) (Mean Log_2_(PC/NT) = 1.9). More than 50% of PC cases (20/39) exhibited high level of CXCL5 expression (Log_2_ (PC/NT) ≥ 1), Figure 1A). We also examined the expression of CXCL5 in PC tissues (*n*=40) and normal penile tissues (*n*=30) using immunohistochemistry. The results showed that the CXCL5 expression was considerably higher in PC tissues than in normal penile tissues (*P*=0.005, Figure 1B).

### Preoperative serum CXCL5 levels are significantly elevated in patients with PC

**Figure 2:**
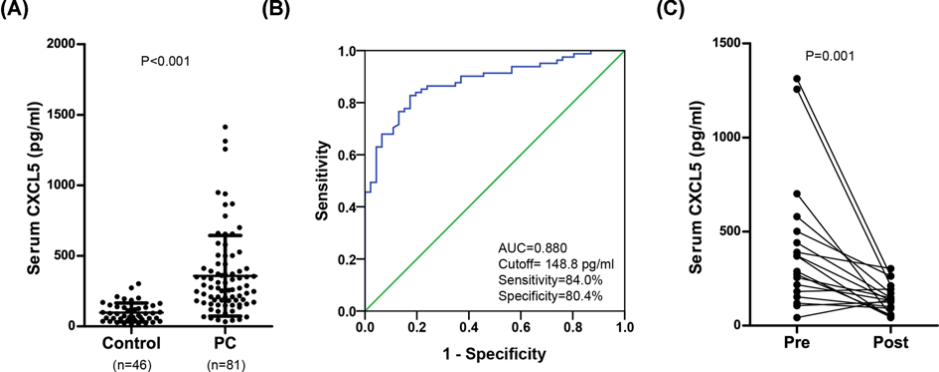
Serum CXCL5 level is highly elevated in PC cohort compared to that in normal male control (**A**) Serum CXCL5 level in preoperative PC cohort (*n*=81) and healthy male control (*n*=46). (**B**) ROC curve analysis of the diagnostic value of serum CXCL5 level in PC patients. (**C**) Serum CXCL5 level in the matched preoperative/postoperative PC cohort (*n*=18).

The finding that CXCL5 was highly expressed in PC tissues prompted us to further investigate the clinical significance of serum CXCL5 in PC. A total of 81 men diagnosed with PC were enrolled in the present study. The detailed summary of the patient and tumor characteristics, including treatment plan, TNM stage, histological subtype and pathological grade are shown in Table 1. Serum CXCL5 levels were measured in healthy male subjects and patients with PC. Preoperative serum CXCL5 levels were significantly higher in the PC cohort (357.9 ± 285.7 pg/ml) compared with that in healthy male control (98.7 ± 66.9 pg/ml; *P*<0.001; Figure 2A). The area under the curve (AUC) was 0.880 with the sensitivity of 84.0%, and specificity of 80.4% to distinguish penile cancer (cutoff = 148.8 pg/ml, Figure 2B). Moreover, serum CXCL5 levels were significantly decreased following PC surgery (*P*=0.001; Figure 2C).

**Table 1:** Clinicopathologic characteristics of PC patient cohort

Parameters	Cases (%)
**Age (year)**	
>52	40 (49.4%)
≥52	41 (50.6%)
Body mass index (kg/m^2^)	
<24	55 (67.9%)
≤24	26 (32.1%)
**Phimosis**	
Yes	64 (79.0%)
No	17 (21.0%)
**Penile surgery**	
Penile preservation	20 (24.7%)
Partial penectomy	56 (69.1%)
Radical penectomy	5 (6.2%)
**Pathological grade**	
G1	52 (64.2%)
G2	24 (29.6%)
G3	5 (6.2%)
**Histological subtype**	
Usual	52 (64.2%)
Papillary	6 (7.4%)
Warty	13 (16.0%)
Verrucous	10 (12.3%)
**T stage**	
T1	45 (55.6%)
T2	31 (38.3%)
T3	5 (6.2%)
**Nodal status**	
Negative	48 (59.3%)
Positive	33 (40.7%)
**Pelvic LNM**	
M0	76 (93.8%)
M1	5 (6.2%)
**Inguinal lymphadenectomy**	
No	46 (56.8%)
Yes	35 (43.2%)

### Preoperative serum CXCL5 levels are associated with tumor progression and unfavorable clinical outcome

**Figure 3:**
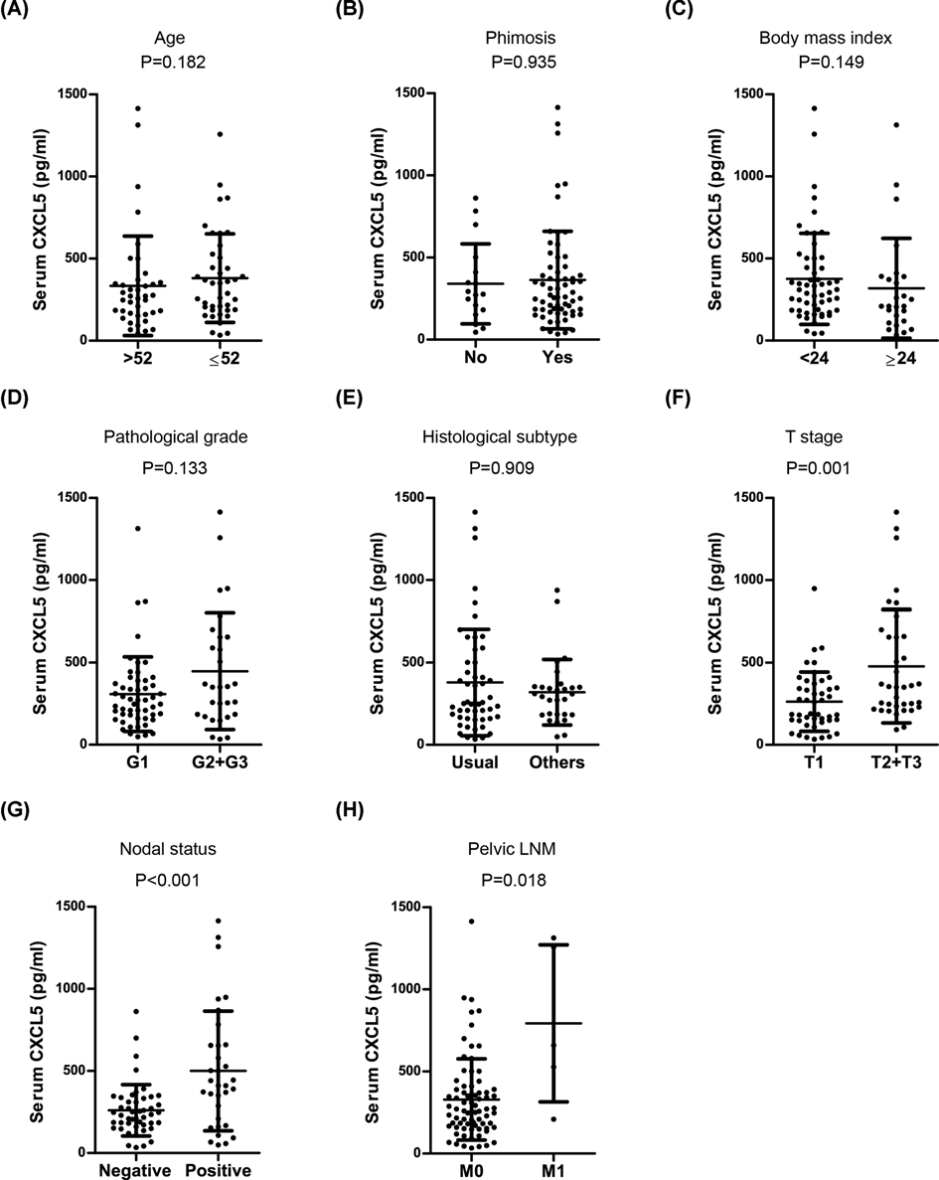
Association of preoperative serum CXCL5 levels with clinical parameters (**A**) Age, (**B**) Phimosis, (**C**) BMI index, (**D**) Pathological grade, (**E**) Histological subtype, (**F**) T stage, (**G**) Nodal status, (**H**) Pelvic LNM.

**Figure 4:**
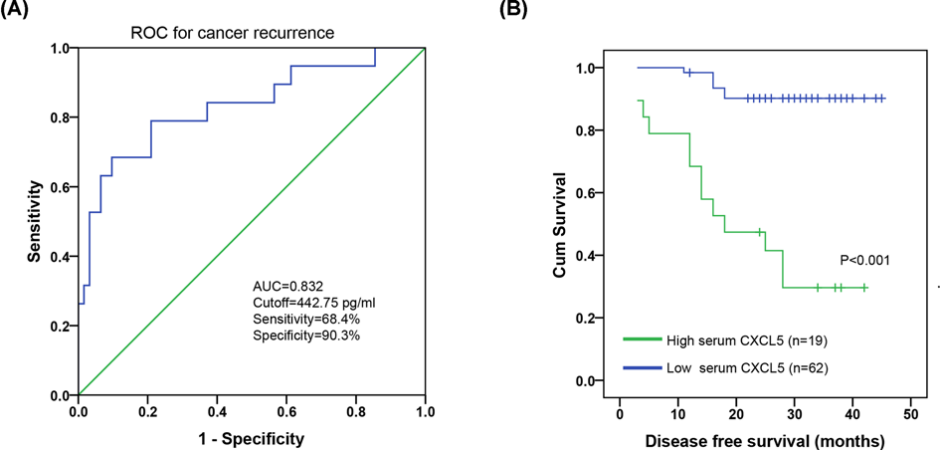
Serum CXCL5 level is associated with unfavorable prognosis in PC (**A**) ROC curves for preoperative serum CXCL5 levels with reference to cancer recurrence. (**B**) PC patients with high preoperative serum CXCL5 levels exhibited shorter DFS.

The association between preoperative serum CXCL5 levels and clinicopathological parameters (age, BMI, pathological grade, phimosis, histological subtype, tumor stage, and nodal status) was analyzed. As shown in Figure 3, preoperative serum CXCL5 levels were significantly associated with oncological parameters including T stage (*P*=0.001), nodal status (*P*<0.001), and pelvic lymph node metastasis (LNM) (*P*=0.018); however, it was not significantly associated with BMI (*P*=0.149), phimosis (*P*=0.935), age (*P*=0.182), histological subtype (*P*=0.909), and pathological grade (*P*=0.133). In addition, ROC analysis showed that CXCL5 had a sensitivity of 68.4% and a specificity of 90.3% to discriminate cancer recurrence (cutoff value: 442.75 pg/ml; AUC, 0.832; Figure 4A). Survival analysis showed that patients with high serum CXCL5 levels exhibited shorter DFS (*P*<0.001) (Figure 4B).

Univariable Cox regression analysis revealed that nodal status (*P*<0.001), T stage (*P*=0.015), pelvic LNM (*P*<0.001), and higher preoperative serum CXCL5 levels (*P*<0.001) were associated with shorter disease-free survival (DFS) in the PC cohort (Table 2). Meanwhile, multivariable Cox regression analysis indicated that nodal status (*P*=0.001; HR: 12.657), pelvic LNM (*P*=0.002; HR: 15.295), and higher preoperative serum CXCL5 levels (*P*=0.001; HR: 6.363) could serve as independent prognostic factors for DFS (Table 2).

**Table 2:** Cox univariate and multivariate proportional hazard model for factors affecting disease-free survival in PC cases

Clinical parameters	Univariate analysis	Multivariate analysis	
	*P* value	HR(95%CI)	*P* value
**T stage** (T1 vs. T2+T3)	0.015		0.320
**Pathological grade** (G1 vs. G2+G3)	0.268		
**Histological subtype** (Usual vs. Others)	0.318		
**Nodal status** (Negative vs. Positive)	<0.001	12.657 (2.782–57.587)	0.001
**Pelvic LNM** (No vs. Yes)	<0.001	15.295 (2.655–88.113)	0.002
**High serum CXCL5** (≥442.75 pg/ml vs. <442.75 pg/ml)	<0.001	6.363 (2.185–18.531)	0.001

### CXCL5 is differentially expressed in PC cell lines and culture supernatants

**Figure 5:**
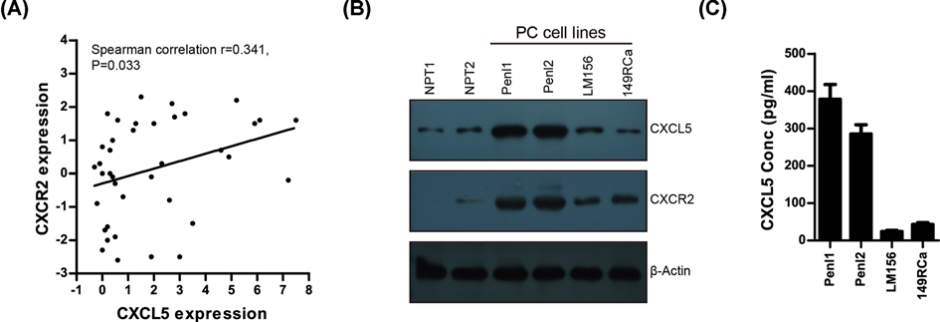
Correlation of gene expression between CXCL5 and CXCR2 in PC tissues (**A**) Correlated expression between CXCL5 and CXCR2 in GSE57955 dataset. (**B**) Expression of CXCL5 and CXCR2 in normal penile tissues (NPT1, NPT2) and PC cell lines. (**C**) CXCL5 secretion in culture supernatant of PC cell lines.

The clinical relevance of CXCL5 expression and its receptor CXCR2 in PC tissues was analyzed in GSE57955 dataset. As shown in Figure 5A, PC cases with high CXCL5 expression also tended to exhibit high CXCR2 expression (spearman correlation *r*=0.341, *P*=0.033). We also observed differential expression of CXCL5 and its receptor CXCR2 in normal penile tissues (NPT1, NPT2) and a panel of PC cell lines (Penl1, Penl2, 149RCa, LM156) (Figure 5B and Supplementary Figure S1A). Consistently, ELISA analysis showed that high CXCL5 level was detected in culture supernatant from PC cell lines exhibiting high endogenous CXCL5 expression (Penl1, Penl2; Figure 5C).

### Knockdown of CXCL5 attenuates malignant phenotype in PC cell lines

**Figure 6:**
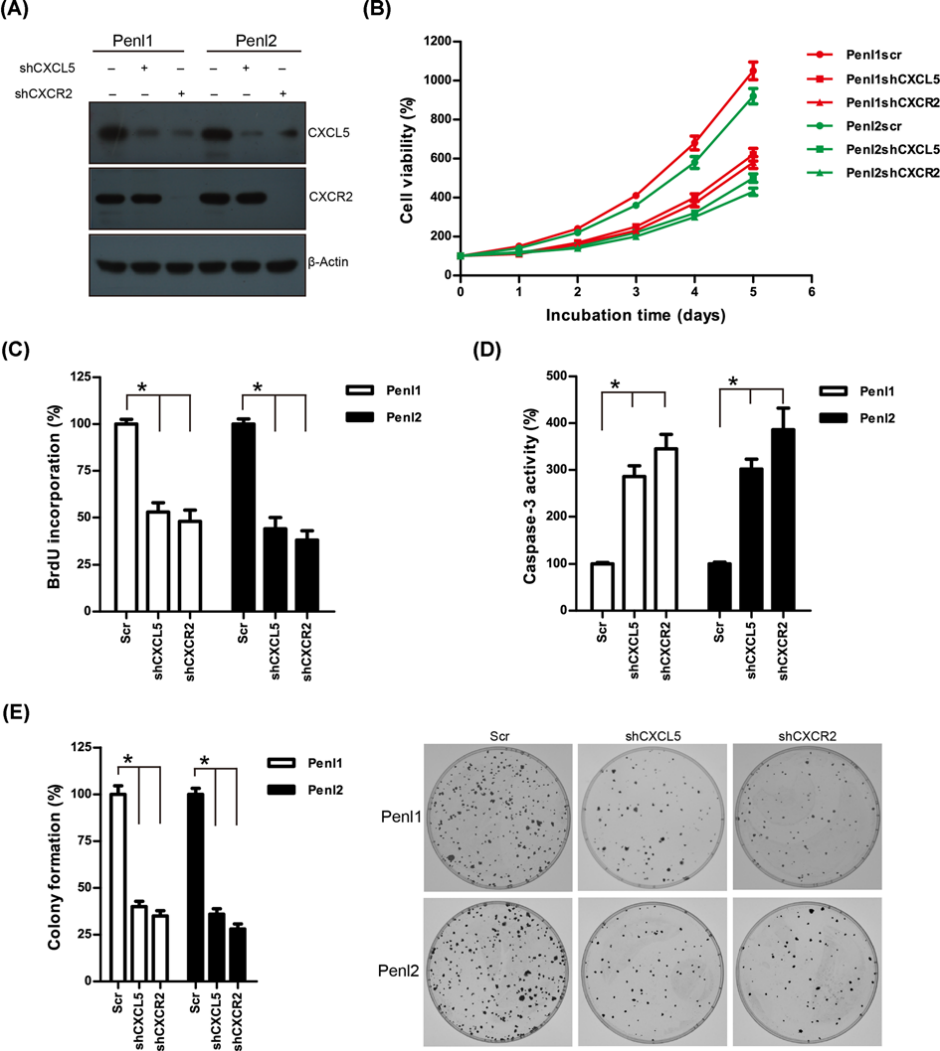
Knockdown of CXCL5 or CXCR2 suppresses cell proliferation, impairs clonogenesis, and induces caspase-3 activity in PC cells (**A**) Western blotting analysis on CXCL5 or CXCR2 expression following shRNA-mediated knockdown in Penl1 and Penl2 cells. (**B**) Depletion of CXCL5 or CXCR2 expression suppressed cell growth of PC cells. (**C**) Knockdown of CXCL5 or CXCR2 reduced BrdU incorporation in PC cell lines. The BrdU incorporation in Scr control was regards as 100%; *n*=3, *P<0.05. (**D**) Knockdown of CXCL5 induced caspase-3 activity in Penl1 and Penl2 cells. The caspase-3 activity in Scr control was regards as 100%; *n*=3, *P<0.05. (**E**) Depletion of CXCL5 expression reduced clonogenesis of PC cells. The colony formed in Scr control was regards as 100%; *n*=3, *P<0.05.

**Figure 7:**
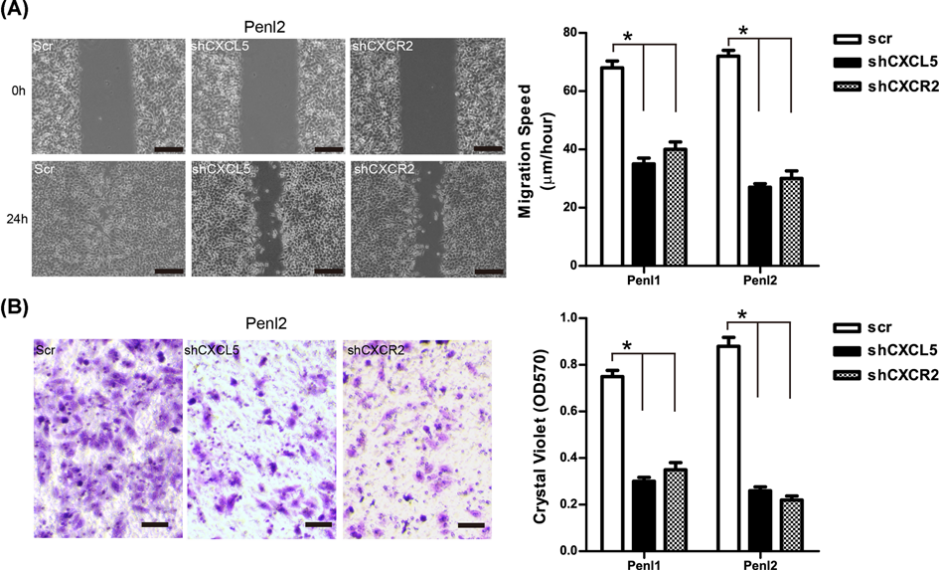
Knockdown of CXCL5 or CXCR2 inhibits cell migration and invasion in PC cells (**A**) Knockdown of CXCL5 or CXCR2 expression inhibited cell migration of PC cells; bars: 100 µm. The cell migration in Scr control was regards as 100%; *n*=3, *P<0.05. (**B**) Knockdown of CXCL5 or CXCR2 expression inhibited transwell invasion of PC cells; bars: 50 µm. The cell invasion in Scr control was regards as 100%; *n*=3, *P<0.05.

The oncogenic function of CXCL5/CXCR2 signaling in the PC Penl1 and Penl2 cell lines was further investigated. Endogenous CXCL5 or CXCR2 expression in Penl1 and Penl2 cells was considerably reduced by shRNAs compared with scramble (Scr) control (Figure 6A and Supplementary Figure S1B). CCK-8 assay revealed that shCXCL5 (cell doubling time, 41.6 ± 1.8 and 48.0 ± 2.1 h for Penl1 and Penl2, respectively) or shCXCR2 (cell doubling time, 42.7 ± 1.2 and 52.2 ± 2.3 h for Penl1 and Penl2, respectively) transfected-PC cells grew slower compared with that in cells transfected with the Scr control (cell doubling time, 34.5 ± 2.2 and 36.0 ± 1.8 h for Penl1 and Penl2, respectively) (*P*<0.05; Figure 6B). In addition, reduced BrdU incorporation was observed in CXCL5 or CXCR2 knockdown Penl1 and Penl2 cell lines, while caspase-3 activity was increased following CXCL5 or CXCR2 knockdown in Penl1 and Penl2 cell lines (*P*<0.05; Figure 6C,D). Colony formation was decreased in the shCXCL5 or shCXCR2 groups compared with that in Scr control group in the Penl1 and Penl2 cell lines (*P*<0.05; Figure 6E). We also overexpressed CXCL5 in CXCL5-low PC cell line LM156 and 149RCa. However, the expression of CXCR2 still remained low following CXCL5 overexpression (Supplementary Figure S2A). Consequently, we detected mild change of cell proliferation and clonogenesis in these two PC cell lines (Supplementary Figure S2B, S2C, S2D). Wound healing assay revealed that cell migration was reduced in shCXCL5 or shCXCR2 groups compared with that in the Scr control group (*P*<0.05; Figure 7A and Supplementary Figure S3A). Furthermore, transwell invasion assay revealed that knockdown of CXCL5 or CXCR2 expression inhibited the invasiveness of PC cells compared with that in the Scr control group (*P*<0.05; Figure 7B and Supplementary Figure S3B).

### CXCL5/CXCR2 regulates downstream STAT3 and AKT signaling and MMP2/9 secretion in PC cell lines

**Figure 8:**
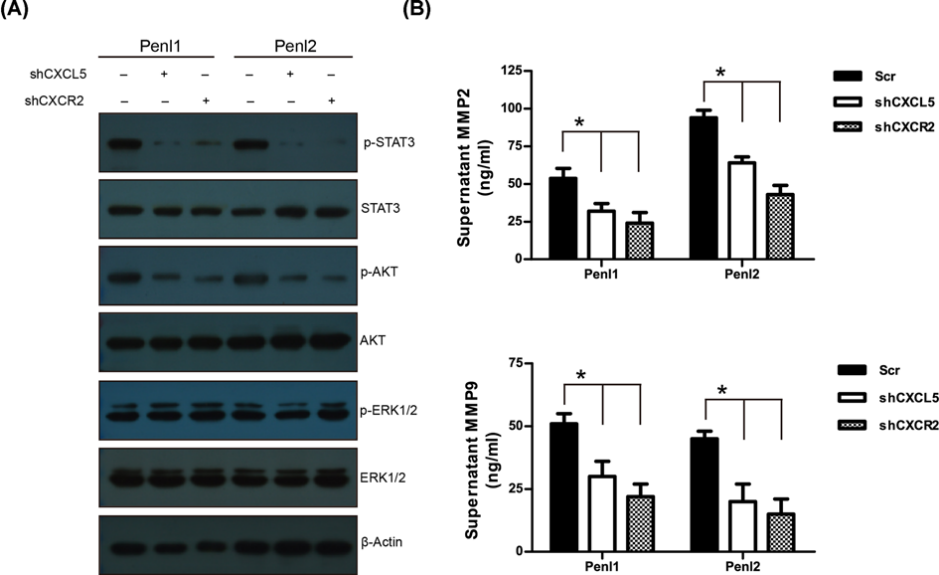
Knockdown of CXCL5 or CXCR2 attenuates STAT3 and AKT signaling and reduces MMP2/9 secretion in PC cells (**A**) Effect of CXCL5 or CXCR2 depletion on cancer-related signaling pathways in PC cell lines. β-Actin was used as a loading control. (**B**) Knockdown of CXCL5 or CXCR2 reduced MMP2/9 secretion in PC cell lines; *n*=3, *P<0.05.

The cancer-related signaling pathways such as PI3K/AKT, ERK1/2, and STAT3 were analyzed using Western blotting. Knockdown of CXCL5 or CXCR2 expression reduced p-STAT3 and p-AKT levels in Penl1 and Penl2 compared with that in the scramble (Scr) control (Figure 8A and Supplementary Figure S4). The levels of p-ERK1/2 remained unchanged following knockdown of CXCl5 or CXCR2 in the Penl1 and Penl2 cell lines (Figure 8A). Meanwhile, ELISA assay revealed that depletion of CXCL5 or CXCR2 reduced secretion of two invasion/metastasis-related molecules MMP2 and MMP9, as compared with Scr control (*P*<0.05) (Figure 8B).

## Discussion

CXCL5 has been found to be involved in tumor progression in numerous types of cancers and could serve as a potential serum biomarker in various malignancies, including breast, nasopharyngeal, gastric, colorectal, and biliary tract cancer [29]. Zhang et al. [30] showed that serum CXCL5 levels could serve as a prognostic biomarker in nasopharyngeal carcinoma, while Lim et al. [31] indicated that serum CXCL5 levels could serve as potential biomarkers to predict the distant metastasis of primary gastric cancer, and Kawamura et al. [32] identified serum CXCL5 levels as a potential prognostic biomarker for colorectal cancer. Recently, Lee et al. [33] observed that serum CXCL5 levels could predict the unfavorable prognosis in advanced biliary tract cancer. The results of the present study found that the expression of tissue and serum CXCL5 levels were increased in PC compared with that in healthy control samples. Moreover, preoperative serum CXCL5 levels were also significantly associated with clinical parameters, including T stage, nodal status, and pelvic LNM in PC; high preoperative serum CXCL5 levels were an independent prognostic factor for DFS in PC. These findings found that preoperative serum CXCL5 levels were associated with tumor progression and could serve as potential diagnostic and prognostic cancer biomarker for PC. However, the possible mechanisms leading to the up-regulation of CXCL5 in PC still remains unknown, despite previous studies on other cancers revealing that CXCL5 expression might be driven by multiple cancer-associated pathways, including nuclear factor-κB and cyclooxygenase-2/prostaglandin E2 [34,35]. Due to the limitations of the present retrospective study (including from a single center, a small cohort, relatively short follow-up period and diversity of treatment), a multicenter prospective study would be required to further validate the potential value of serum CXCL5 as a biomarker for PC.

CXCL5 could regulate tumor progression in an autocrine or paracrine manner in a vast number of cancers. Gao et al. [36] found that autocrine CXCL5/CXCR2 signaling could promote the migration and invasion of bladder cancer cells, while Zhou et al. [20] revealed that autocrine CXCL5/CXCR2 signaling could enhance epithelial–mesenchymal transition in hepatocellular carcinoma cells. Moreover, paracrine CXCL5 secreted by cancer-associated stromal cells (mesenchymal stem cells and macrophages) could also promote cancer cell invasion and dissemination [37,38]. The results from the present study revealed aberrant expression of CXCL5 in PC tissues, cell lines, and their culture supernatants. Moreover, consistent expression of CXCL5 and CXCR2 was also observed in PC tissues and cell lines, suggesting CXCL5 could act in an autocrine manner in PC. Furthermore, knockdown of CXCL5 or CXCR2 attenuated cell proliferation, clonogenesis, migration/invasion and induced apoptosis in PC cell lines, suggesting autocrine CXCL5/CXCR2 signaling axis might be crucial to promote cell proliferation, tumorigenicity, migration/invasion and apoptosis escape in PC. Several CXCR2 inhibitors (AZD5069, SB225002, SCH-527123, and danirixin) are currently under development for cancer treatment [39]. Experimental therapeutics on CXCL5/CXCR2 inhibitors would be the priority of future studies in penile cancer.

The CXCL5/CXCR2 axis could activate multiple downstream signaling pathways, including PI3K/AKT, ERK1/2, and STAT3 to promote tumor progression in cancers. CXCR2/CXCL5 axis could activate PI3K/AKT signaling in hepatocellular carcinoma cells [40] and also activate the STAT3 signaling pathway to promote the migration and invasion in gastric cancer [37]. Hsu et al. [41] demonstrated that CXCL5 could increase ERK1/2 activation during the tumor progression of breast cancer. In the present study, knockdown of CXCL5 or CXCR2 attenuated downstream AKT and STAT3 signaling pathways and reduced MMP2/9 secretion in PC cell lines. As AKT and STAT3 pathways were proven to be important for PC tumorigenesis [42,43], it would be reasonable to propose that CXCL5/CXCR2 signaling might activate AKT and STAT3 signaling pathways to promote tumor progression in PC. However, the attenuation of ERK1/2 signaling following knockdown of CXCL5 or CXCR2 in the PC cell lines was not observed, suggesting that CXCL5/CXCR2 signaling might activate differential downstream signaling dependent in specific cancer types.

In conclusion, high preoperative serum CXCL5 levels were associated with PC progression and could serve as a potential prognostic biomarker for PC. Furthermore, the CXCL5/CXCR2 axis might be required for PC progression through activating AKT and STAT3 signaling pathway and inducing MMP2/9 secretion. New discoveries in the CXCL5/CXCR2 signaling would also aid clinical decision-making for PC patients, bringing us closer to the promise of translational precision medicine.

## Supplementary Material

Supplementary Figures S1-S4

## Data Availability

All data generated or analyzed during this study are included in this published article.

## Abbreviations

BrdU: bromodeoxyuridine
CA: cancer antigen
CCK-8: Cell Counting Kit-8
CEA: carcinoembryonic antigen
CXCL5: C-X-C motif chemokine ligand 5
DFS: disease-free survival
IHC: immunohistochemistry
LNM: lymph node metastasis
PC: penile cancer
ROC: receiver-operating characteristic
SCC: squamous cell carcinoma antigen
