# Supplementary material for: Serum CXCL5 level is associated with tumor progression in penile cancer
Source: Biosci Rep. 2021 Jan 27;41(1):BSR20202133. doi: 10.1042/BSR20202133 (PMC7843497; doi:10.1042/BSR20202133)
Supplement: Supplementary Figures S1-S4 [file BSR-2020-2133_supp.pdf]

Supplementary Figures

Figure S1

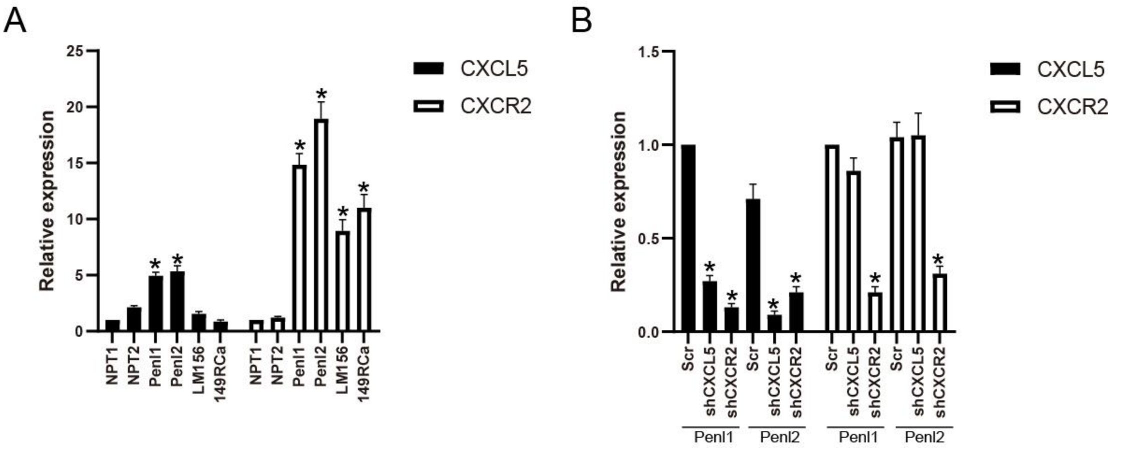

Figure S1. Densitometric analysis and subsequent statistical analysis for **Fig 5B and Fig 6A**.**A.**Densitometric analysis and subsequent statistical analysis for Fig. 5B. *P<0.05, as compared with NPT1 and NPT2. **B.**Densitometric analysis and subsequent statistical analysis for Fig. 6A. *P<0.05, as compared with Scr control in Penl1 or Penl2, respectively.

Figure S2

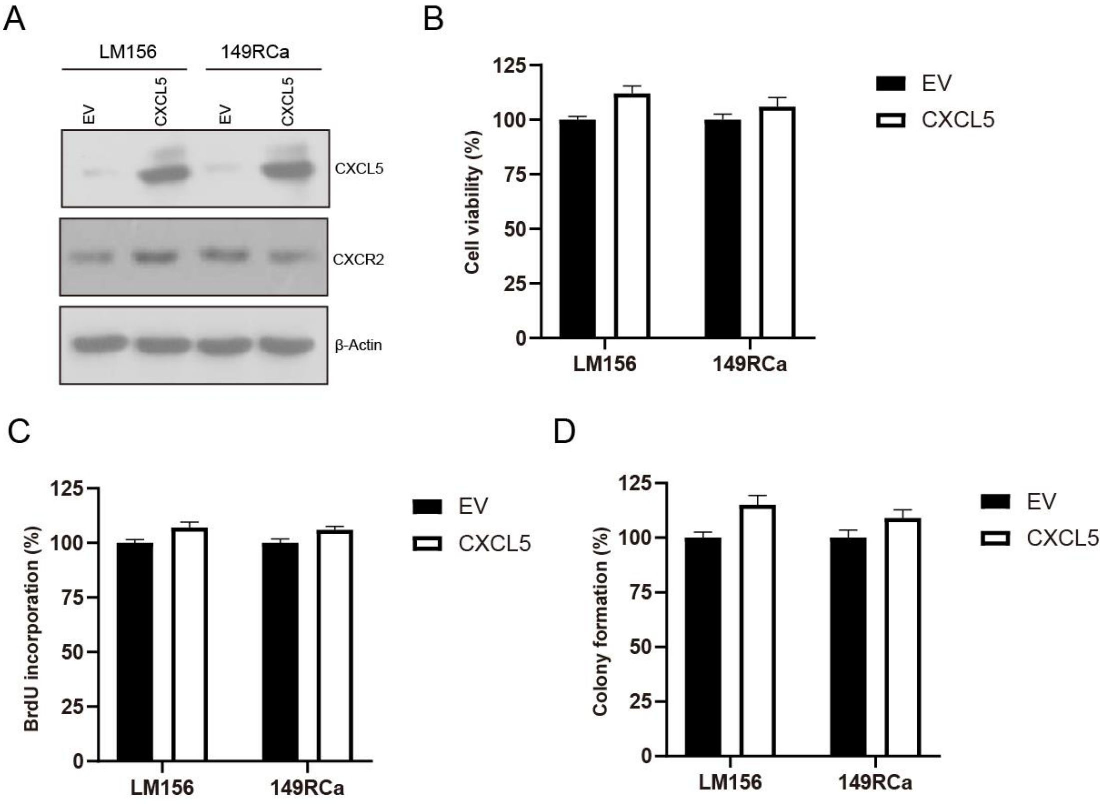

Figure S2. Over-expression of CXCL5 mildly affected cell proliferation and clonogenesis in in LM156 and 149RCa cells. **A**. Over-expression of CXCL5 in LM156 and 149RCa cells. **B.** Over-expression of CXCL5 mildly affected cell growth of PC cell lines. The cell viability in empty vector (EV) control was regards as 100%.**C.**Over-expression of CXCL5 mildly affectedBrdU incorporation in PC cell lines. The BrdU incorporation in empty vector (EV) control was regards as 100%.**D**. Over-expression of CXCL5 mildly affected colony formation in LM156 and 149RCa cells.The colony formation in empty vector (EV) control was regards as 100%.

Figure S3

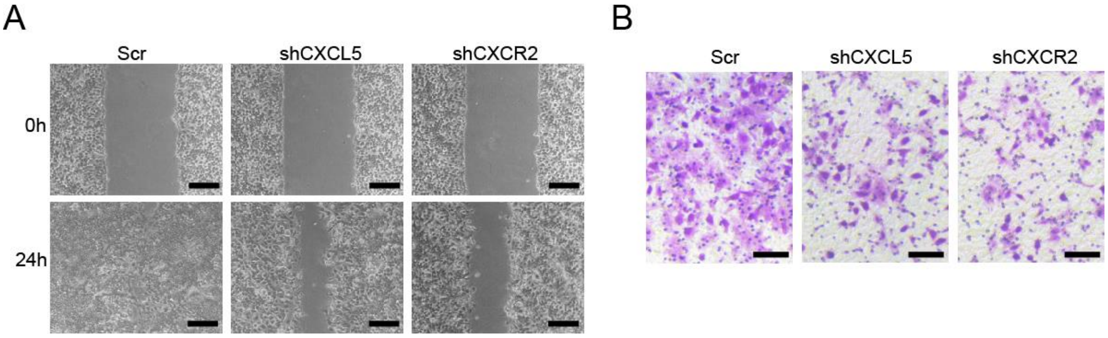

Figure S3. Knockdown of CXCL5 or CXCR2 expression inhibited cell migration and transwell invasion ofPenl1 cells**A**. Knockdown of CXCL5 or CXCR2 expression inhibited cell migration ofPenl1 cells. Bars: 100 μm.**B.**Knockdown of CXCL5 or CXCR2 expression inhibitedtranswell invasion ofPenl1 cells. Bars: 50 μm.

Figure S4

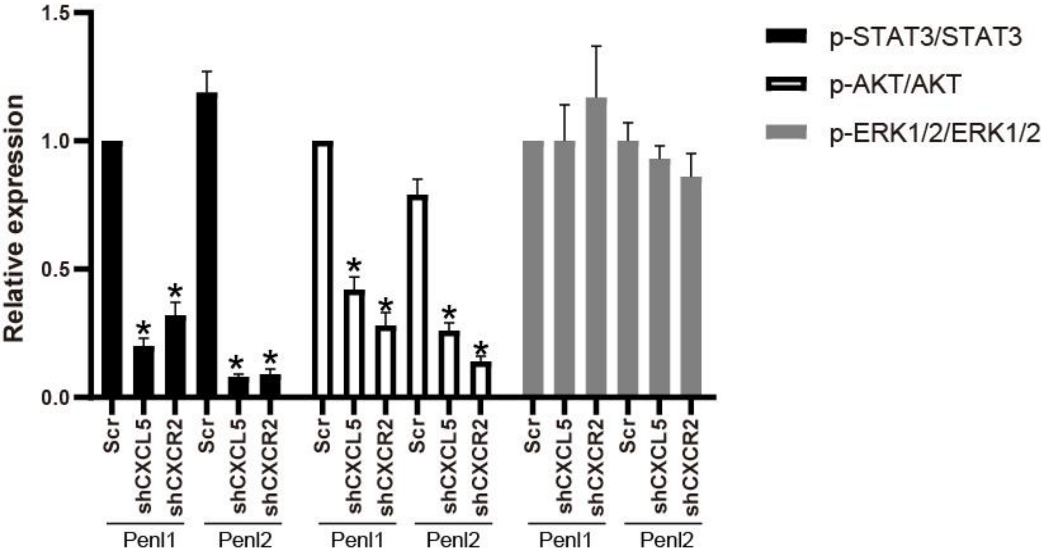

Figure S4. Densitometric analysis and subsequent statistical analysis for **Fig. 8A**. *P<0.05, as compared with Scr control in Penl1 or Penl2, respectively.
